# A104 INVISIBLE COLONIC MALIGNANCY AND POSSIBLE IDIOSYNCRATIC DRUG-INDUCED LIVER INJURY FROM VEDOLIZUMAB IN A PATIENT WITH ULCERATIVE COLITIS, PRIMARY SCLEROSING CHOLANGITIS AND AUTOIMMUNE HEPATITIS OVERLAP SYNDROME

**DOI:** 10.1093/jcag/gwac036.104

**Published:** 2023-03-07

**Authors:** G Malhi, M Cheah, A Wilson, K Qumosani, R Khanna

**Affiliations:** 1 Gastroenterology, Western University, London; 2 Gastroenterology, Scarborough Health Network, Toronto, Canada

## Abstract

**Background:**

Inflammatory bowel disease (IBD) is characterized by chronic inflammation of the gastrointestinal tract. Due to an increased risk of developing colorectal cancer, regular surveillance for dysplastic lesions via colonoscopy is recommended. Invisible dysplasia is the abnormal development of cells noted on pathology with no visible lesion seen during colonoscopy. Primary sclerosing cholangitis (PSC) is an immune-mediated liver disease leading to progressive stricturing and fibrosis of the bile ducts. There is significant association between PSC and IBD, as up to 80% of patients with PSC having underlying IBD. Drug induced liver injury (DILI) is a common cause of acute liver failure in most Western countries. Most cases of DILI are self-limited, with resolution of laboratory and clinical findings after cessation of the offending agent.

**Purpose:**

To describe a case of invisible colonic malignancy and possible idiosyncratic drug-induced liver injury from vedolizumab (VDZ) in a patient with ulcerative colitis (UC), PSC and autoimmune hepatitis (AIH) overlap syndrome.

**Method:**

Patient consent was obtained. Information from electronic records was extracted, including admission notes and procedural reports. A literature review was performed using Pubmed.

**Result(s):**

A 32-year-old Caucasian female with UC on VDZ with PSC and AIH had multiple colonoscopies performed demonstrating multifocal low-grade dysplasia. However, she remained resistant to surgery. She presented to clinic with tea-coloured urine, fatigue, and scleral icterus. Investigations revealed conjugated hyperbilirubinemia with elevation in hepatocellular liver enzymes. Imaging was consistent with large duct PSC, and liver biopsy showed grade 2 chronic hepatitis raising the possibility of large bile duct obstruction. She underwent liver transplant assessment and VDZ was held. Her bilirubin and liver enzymes recovered. Given multiple colonoscopies showing multifocal dysplastic changes and the patient declining other biologics, she would ultimately undergo total proctocolectomy with end ileostomy. Pathology demonstrated mucinous adenocarcinoma with a signet ring component.

**Conclusion(s):**

Both mucinous adenocarcinoma and signet ring carcinoma are more aggressive malignancies associated with poor prognosis. Found more often in younger patients, they are often diagnosed in later stages with lymphovascular invasion. Here, there was no evidence of metastases in any of the 40 lymph nodes examined, which indicated more favourable prognosis.

The mechanism by which VDZ potentially causes liver injury is unknown. Multiple therapies for PSC-AIH overlap and IBD were entertained as causative agents. However, significant improvement in clinical symptoms and serologic parameters following cessation of VDZ was temporally suggestive.

This case simultaneously highlights the importance of both timely colectomy in IBD patients with high-risk features during surveillance and early recognition and cessation of potential causative medications which induce liver injury.

**Please acknowledge all funding agencies by checking the applicable boxes below:**

None

**Disclosure of Interest:**

None Declared

